# Prostate cancer involving bilateral seminal vesicles along with bone and testicular metastases: a case report

**DOI:** 10.1186/s13256-017-1551-5

**Published:** 2018-03-09

**Authors:** Qingqiang Gao, Jianhuai Chen, Yutian Dai

**Affiliations:** Department of Andrology, Nanjing Drum Tower Hospital, The Affiliated Hospital of Nanjing University Medical School, Nanjing, 210008 China

**Keywords:** Prostate cancer, Testicular metastases

## Abstract

**Background:**

In the past 20 years, the incidence of prostate cancer has risen rapidly. It has been ranked as the third most common malignant tumor of the male genitourinary system. Testicular metastasis is uncommon in prostate cancer. Most cases are incidentally found in the treatment of prostate cancer with orchiectomy. Therefore, we believed it was necessary to report the case of our patient with this disease.

**Case presentation:**

We present a case of a 69-year-old Han Chinese man with a high total prostate-specific antigen level. A transrectal ultrasound-guided prostate biopsy was performed. A pathology report showed prostate cancer tissue with a Gleason score of 4 + 4 = 8/10. Imaging findings suggested that the prostate cancer tissue involved bilateral seminal vesicles and multiple bones. Next, radioactive seed implantation was carried out, and endocrine therapy was continued after the operation. Then enlargement of the left scrotum was found along with a total prostate-specific antigen level of 19.21 ng/ml. Computed tomography of the middle abdomen and pelvic cavity revealed 2.0 × 1.3-cm lesions of the left testis. The patient underwent a left testicular high resection and right orchiectomy. The postoperative pathology report showed metastatic prostate cancer cells in the left testis.

**Conclusions:**

Testicular metastasis of prostate cancer is rare. Therefore, a testicular physical examination is necessary for patients without relapse to avoid a missed diagnosis. Testicular metastasis should be treated according to the principle of treatment for advanced prostate adenocarcinoma if testicular metastasis of prostate adenocarcinoma is detected.

## Background

Prostate cancer is a common malignancy among older men, and it is the sixth leading cause of cancer-related death in men. Moreover, the occurrence of metastatic disease is a major morbidity of prostate cancer. Advances in therapies for prostate cancer have improved survival of men with metastatic prostate cancer. However, testicular metastasis from prostate cancer is rare, and early diagnosis is difficult. Early diagnosis could result in a reduction in the number of men with metastatic prostate cancer.

A man was admitted to our hospital with prostate cancer involving bilateral seminal vesicles and with bone and testicular metastases. Our observations provided a clear basis that testicular examination was necessary for prostate cancer to avoid a missed diagnosis. This case report presents the pathology, diagnosis, and treatment of this disease according to the medical literature to improve the understanding of prostate cancer.

## Case presentation

Our patient was a 69-year-old Han Chinese man. He had no past medical history of hypertension, hyperlipidemia, coronary heart disease, insulin-dependent diabetes mellitus, renal disease, or surgeries. Moreover, he did not smoke and did not consume alcohol, and he had no family history of prostate cancer. He had not received any medical treatment prior to diagnosis. After his medical history was completed, we performed a physical examination, including blood pressure, weight, and height. The patient’s blood pressure was normal, his heart and lungs were normal, and his abdomen was soft. His laboratory test results were normal, including routine blood analysis, urinalysis, liver function tests, kidney function tests, and an electrocardiogram.

In 2013, the patient had been found to have high levels of total prostate-specific antigen (TPSA) (8.43 ng/ml) and free prostate-specific antigen (FPSA) (0.752 ng/ml). However, the increased levels of TPSA and FPSA did not draw enough attention to the patient, and no special treatment was given. TPSA > 100.0 ng/ml and FPSA of 18.41 ng/ml were found in July 2015. We were told at that time that the patient had refused a digital rectal examination (DRE) in 2013 by telephone follow-up. No obvious frequent micturition, urinary urgency, dysuria, or gross hematuria was found.

The patient was admitted to Nanjing Gulou Hospital on 14 July 2015. After admission, his TPSA level increased to > 100.0 ng/ml, his FPSA level was 18.41 ng/ml, and his prostate acid phosphatase (PAP) level was 18.30 ng/ml. A transrectal ultrasound-guided prostate biopsy was performed on 17 July 2015. The pathology report indicated that cancer tissues were found in 2, 3, 5, 7, 8, 9, 10, 11, and 12 points of prostate. The patient’s Gleason score was 4 + 4 = 8/10. Magnetic resonance imaging findings suggested that prostate cancer involved bilateral seminal vesicles and multiple pelvic lymph nodes. Findings of computed tomography (CT) suggested a high-density shadow in cervical vertebra C7, lumbar vertebra L2, the left sixth rib, and the left ilium. The results of emission computed tomography suggested that lesions were located in the second lumbar vertebra, right rear fifth rib, and right parietal bone.

The following endocrine therapy was provided: triptorelin embonate by subcutaneous injection every 28 days and bicalutamide 50 mg once daily. The patient’s Gleason score was high, his prostate was hard, and fixation was detected by DRE. Moreover, shrinkage of the lesion was demonstrated after the patient received endocrine treatment. Considering the concomitant disease with paroxysmal supraventricular tachycardia, the patient was not suitable for radical prostatectomy. Therefore, we believed that treatment with radioactive seed implantation would be beneficial. Radioactive seed implantation was performed with guidance by B-mode ultrasound on 20 October 2015. Endocrine therapy was continued after the operation. Enlargement of the left scrotum along with sensation of scrotum tenesmus was found in April 2017. However, no obvious pain, chills, fever, frequent micturition, urinary urgency, or dysuria was found.

A 2.0-cm uneven echo and a 1.2 × 0.9-cm heterogeneous hyperecho were observed in the left testis by color Doppler ultrasound on 26 April 2017. The boundary was not clear, and the echo was uneven, which suggested that the echo area might show benign lesions. For further diagnosis and treatment, the patient was readmitted to the hospital on 5 May 2017. His TPSA level was 19.21 ng/ml, and his testosterone level was 1.02 mmol/L. The results of CT and enhanced CT of the middle abdomen and pelvic cavity revealed an approximately 2.0 × 1.3-cm mixed-density shadow in the left testis, which showed obviously inhomogeneous strengthening, and a high-density shadow in the L2 vertebral body and left iliac bone.

The patient underwent a left testicular high resection and right orchiectomy on 11 May 2017. The postoperative pathology report showed metastatic prostate cancer tissue in the left testis. The patient’s Gleason score was 5 + 5 = 10/10. His World Health Organization/International Society of Urological Pathology classification was 5/5. The tumor size was 1.3 × 1.2 × 1 cm. The cancer tissue did not penetrate the tunica albuginea testis. Tumor emboli were found in the vasculature. No nerve was involved by the cancer tissue. No cancer tissue was left in the left epididymis and spermatic cord. No cancer tissue was found in the right testis and epididymis tissue. The results of immunohistochemistry indicated the following cancer cell expression: prostate-specific antigen (PSA)-positive, prostate-specific acid phosphatase-positive, CR-negative, CD99-negative, prostate inhibin peptide-negative, erythroblast transformation-specific-related gene (ERG)-negative, placental alkaline phosphatase (PLAP)-negative, CD117 weakly positive, SA114-negative, organic cation transporter 2 (OCT2)-negative, and Ki-67 > 40% positive.

**Fig. 1:**
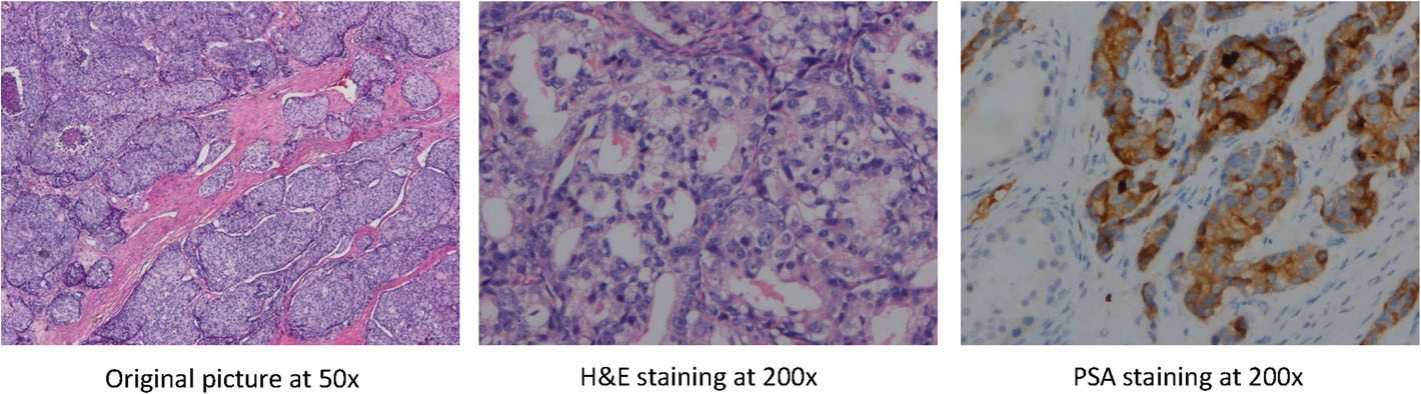
Postoperative left testicular pathological tissue. *PSA* Prostate-specific antigen

The patient’s TPSA level after the operation was 13.59 ng/ml, and his FPSA level was 2.20 ng/ml, on 29 June 2017. His testosterone level after the operation was 1.02 mmol/L on 13 May 2017. The patient was still receiving the combined androgen blockade and endocrine therapy as of December 2017. The patient is receiving orally administered abiraterone 1000 mg/day and prednisone 10 mg/day for 3 months. His TPSA level was 30–40 ng/ml at the time of this writing (Fig. 1).

## Discussion

Although testicular metastasis of prostate cancer is rare, metastatic disease is a major morbidity of prostate cancer. On the basis of the case of our patient, we believe that testicular examination is necessary for patients with prostate cancer to reduce the incidence of metastatic prostate cancer.

The incidence of testicular metastasis in prostate cancer has been reported to be about 0.06% [1]. Unilateral metastasis is more common [2]. Testicular metastasis was reportedly found in about 4% cases of orchiectomy for prostate cancer [3]. No lumps, pain, or hydroceles were found in previously reported patients, which was confirmed only by histopathological examination [4]. In rare cases, patients have been admitted to the hospital for testicular pain or testicular masses [4]. Patients with prostate cancer with testicular masses should be differentiated from those with primary testicular tumors [5]. Patients with testicular metastasis of prostate cancer are often older than those with primary testicular tumors. PSA and PAP staining are more often positive in patients with testicular metastasis, which could help differentiate patients with testicular metastasis from those with primary testicular tumors. Identifying the source of testicular cancer is critical for tumor staging and treatment.

Testicular metastasis was often considered a manifestation of advanced prostate cancer. Case-control studies about the difference in prognosis between patients with prostate cancer with versus without testicular metastasis are rare. Researchers in one study reported that three patients with testicular metastasis of prostate cancer died within 1 year despite receiving maximal androgen deprivation therapy [6]. Although their Gleason scores were higher, patients with prostate cancer with testicular metastasis were sensitive to endocrine therapy and radiotherapy [7]. The average duration of endocrine therapy was 33 months [7]. Most patients with testicular metastasis of prostate cancer also had other organ metastases, and their PSA levels often increased significantly [6]. However, PSA levels of some patients were low [7]. Therefore, testicular examination is necessary for patients without relapse to avoid a missed diagnosis.

## Conclusions

This case report shows that testicular examination is needed for patients with prostate cancer, especially for patients without relapse in whom the PSA level is low. Therefore, testicular examination could significantly reduce the risk of developing metastatic prostate cancer involving bilateral seminal vesicles and with bone and testicular involvement at the time of diagnosis.
